# A novel multi-locus sequence typing (MLST) protocol for *Leuconostoc lactis* isolates from traditional dairy products in China and Mongolia

**DOI:** 10.1186/1471-2180-14-150

**Published:** 2014-06-09

**Authors:** Tong Dan, Wenjun Liu, Zhihong Sun, Qiang Lv, Haiyan Xu, Yuqin Song, Heping Zhang

**Affiliations:** 1 Key Laboratory of Dairy Biotechnology and Engineering, Education Ministry of P. R. China, Department of Food Science and Engineering, Inner Mongolia Agricultural University, Hohhot 010018, P. R. China

## Abstract

**Background:**

Economically*, Leuconostoc lactis* is one of the most important species in the genus *Leuconostoc*. It plays an important role in the food industry including the production of dextrans and bacteriocins. Currently, traditional molecular typing approaches for characterisation of this species at the isolate level are either unavailable or are not sufficiently reliable for practical use. Multilocus sequence typing (MLST) is a robust and reliable method for characterising bacterial and fungal species at the molecular level. In this study, a novel MLST protocol was developed for 50 *L. lactis* isolates from Mongolia and China.

**Results:**

Sequences from eight targeted genes (*groEL*, *carB*, *recA*, *pheS*, *murC*, *pyrG*, *rpoB* and *uvrC*) were obtained. Sequence analysis indicated 20 different sequence types (STs), with 13 of them being represented by a single isolate. Phylogenetic analysis based on the sequences of eight MLST loci indicated that the isolates belonged to two major groups, A (34 isolates) and B (16 isolates). Linkage disequilibrium analyses indicated that recombination occurred at a low frequency *in L. lactis*, indicating a clonal population structure. Split-decomposition analysis indicated that intraspecies recombination played a role in generating genotypic diversity amongst isolates.

**Conclusions:**

Our results indicated that MLST is a valuable tool for typing *L. lactis* isolates that can be used for further monitoring of evolutionary changes and population genetics.

## Background

*Leuconostoc* species are usually found in association with plant, dairy, meat or other food products. Like other lactic acid bacteria (LAB), *Leuconostoc* species are important industrial starter microbes that are used in several industrial and food fermentation processes, such as the production of cheese, butter, buttermilk, kefir, sourdough and kimchi [1,2]. These species are closely related to heterofermentative species in the genus *Lactobacillus*[3]*.* Phenotypically, the genus *Leuconostoc* and *Lactobacillus* are often isolated from the same habitats and share many characteristics [4].

The genus *Leuconostoc* was first described by Van Tieghem [5]. In recent years, several species have been reclassified within the genus; some new species have been added and new genera have been erected from species previously considered to belong to *Leuconostoc*. For example, the species *L. mesenteroides* was reclassified into three subspecies: *L. mesenteroides* subsp. *mesenteroides*, *L. mesenteroides* subsp. *dextranicum* and *L. mesenteroides* subsp. *cremoris*[6]. A new species, *L. fallax* was identified from sauerkraut [7] and subsequently a number of *L. fallax* isolates have been found in the heterofermentative stage of sauerkraut fermentation [7,8]. The *L. paramesenteroides* group of species have been reclassified into a new genus, *Weisella*[8]; *L. oenos* has been reclassified into the genus *Oenococcus* as *O. oeni*[9] and *L. durionis*, *L. ficulneum*, *L. pseudoficulneum* and *L. fructosum* have been assigned to a new genus, *Fructobacillus*[10]. Furthermore, *L. argentinum* has been reclassified as a synonym of *L. lactis* following numerical analysis of repetitive extragenic palindromic-PCR patterns, whole-cell protein profiles (SDS-PAGE) and fluorescent amplified fragment length polymorphism (FAFLP) band patterns [11]. New species, including L. holzapfelii, *L. palmae* and *L. miyukkimchii*, have also been identified from wine and kimchi [12-14].

Typing methods for intraspecies identification of pathogens are essential epidemiological tools in infection prevention and control [15] and have also been applied to LAB. Typing methods are divided into two major categories i.e., phenotypic and genotypic methods. Traditional phenotyping methods, such as the use of serotypes, biotypes, phage-types and antibiograms, have been used for many years to isolate and characterise LAB and, sometimes, to distinguish between species and subspecies. Compared with phenotypic typing methods, genotypic typing methods have some advantages as they have more general applicability and greater discriminatory power. Currently, several molecular typing approaches, such as random amplified polymorphic DNA (RAPD)-PCR, pulsed-field gel electrophoresis (PFGE), restriction fragment length polymorphism (RFLP), protein fingerprinting*,* and repetitive element palindromic PCR (Rep-PCR), have been used to characterise *Leuconostoc* species [16-23].

Multilocus sequence typing (MLST) is a technique for distinguishing accurately between different isolates within a species. MLST is based on the principles of phenotypic multi-locus enzyme electrophoresis (MLEE). MLEE is a typing method that relies on differences in electrophoretic mobility of different enzymes present within a bacterium [15]. Maiden *et al.,*[24] first used the MLST method to identify virulent lineages of 107 isolates of *Neisseria meningitides,* a naturally transformable Gram-negative pathogenic bacterium [24]. Shortly thereafter, the method was used to analyse nonpathogenic food production bacteria including LAB. For example, Tanigawa and Watanabe [25] used MLST to compare seven housekeeping genes in 41 isolates of *Lactobacillus delbrueckii* and demonstrated that MLST was efficient for identification of isolates to subspecies level [25]. De Las Rivas *et al.*[26] compared the genetic diversity and genetic relationships amongst 18 *O. oeni* isolates using the *gyrB*, *pgm*, *ddl*, *recP* and *mleA* genes and MLST [26]. Bilhère *et al*. [27] found that MLST and pulsed-field gel electrophoresis (PFGE) were both useful for identifying 43 isolates of *O. oeni*, although the MLST method was more efficient [27]*.* Although the population biology of some LAB species has been characterised by MLST methods, to date, there is no MLST protocol available for *Leuconostoc* species.

The aim of the present study was to develop an effective MLST protocol for characterisation of *L. lactis* isolates and use this to explore the population structure and evolutionary relationships amongst isolates of this species.

## Results

### Assignment of sequence types

Fifty *L. lactis* isolates were typed using the MLST protocol. Isolates could be divided into 20 sequence types (STs) using combined data from eight loci. ST14 was the most frequent (21 isolates), followed by ST11 (four isolates), ST3 (three isolates), ST4 (three isolates), ST1 (two isolates), ST8 (two isolates) and ST12 (two isolates); there was only one isolate in each of the remaining 13 STs.

### MLST protocol and allelic variation

Eight genes were successfully sequenced and analysed by MLST for all isolates in this study. Polymorphic sites, guanine-cytosine content, rate of non-synonymous (*d*_*N*_) and synonymous (*d*_*S*_) substitutions and the *d*_*N*_*/d*_*S*_ for each locus (*groEL*, *carB*, *recA*, *pheS*, *murC*, *pyrG*, *rpoB* and *uvrC* ) were determined (Table 1). Fragment sizes of the eight selected loci ranged from 550 bp (*recA*) to 892 bp (*groEL*) (Table 2). The number of polymorphic sites per locus ranged from 3 (*recA*) to 9 (*murC*) and a total of 47 SNPs were identified (Table 1). The mean guanine-cytosine content of the partial sequence of the eight gene fragments ranged from 43.12% (*pyrG*) to 48.31% (*recA*), while it was 37.7% in the whole *L. mesenteroides* subsp. *mesenteroides* ATCC 8293 genome previously described [28]. The value of the non-synonymous (*d*_*N*_) and synonymous (*d*_*S*_) substitutions ranged from 0.0000 (*groEL*) to 0.0077 (*murC*) and 0.0556 (*groEL*) to 0.2852 (*carB*) respectively. The lowest *d*_*N*_*/d*_*S*_ ratio (<1) calculated for all eight loci is suggestive of weak purifying selection pressure.

**Table 1:** Allelic variation in 8 housekeeping genes

**Locus**	**Polymorphic sites**	**GC% content (mol%)**	***d***_***N***_	***d***_***S***_	***d***_***N***_ ***/d***_S_^*^
***carB***	4	44.09%	0.0100	0.2852	0.0349
***groEL***	5	46.24%	0.0000	0.0556	0.0000
***murC***	9	44.90%	0.0077	0.2467	0.0313
***pheS***	5	45.26%	0.0012	0.0900	0.0130
***pyrG***	8	43.12%	0.0016	0.1356	0.0114
***recA***	3	48.31%	0.0025	0.2399	0.0104
***rpoB***	7	43.97%	0.0018	0.0715	0.0245
***uvrC***	6	43.68%	0.0028	0.2684	0.0103

*The ratio of non-synonymous (*d*_*N*_) and synonymous (*d*_*S*_) substitutions is indicative of selective pressure on loci.

**Table 2:** Genes and sequencing primers used

**Gene**	**Protein**	**PCR primers**	**Amplicon size (bp)**	Location*
*pyrG*	CTP synthase	5′-AGCAAACACCCAAGAACG-3′	598	481322 to 482935
		5′-TGGTGAAGCGAAGACAAA-3′		
*rpoB*	DNA-directed RNA polymerase subunit beta	5′-CACTGTGCGGTCGTCTTCC-3′	608	1798123 to 1801731
		5′-GCGTTCTCCTGGTATCTATT-3′		
*groEL*	Chaperonin GroEL	5′-CGGTGATAAGGCTGCTGT-3′	892	1734716 to 1736335
		5′-TTTGTTGGGTCCACGATA-3′		
*recA*	Recombinase A	5′-GGAGTCGTTTCTGGGTTAC-3′	550	555064 to 556221
		5′-GTTGCTTTAGGCGTTGGTG-3′		
*uvrC*	Excinuclease ABC subunit C	5′-AGAAATACAAGCCGTACTACAA-3′	560	483053 to 484852
		5′-TCTTCATCAGCGGAACCAA-3′		
*carB*	Carbamoyl phosphate synthase large subunit	5′-ATGGGTTGTGGGAGTTGTA-3′	833	1202174 to 1205353
		5′-ACTTGTTGCGTCGTGGTGT-3′		
*murC*	UDP-N-acetylmuramate-L-alanine ligase	5′-TTTCATAGGCGAACTCAT-3′	619	679802 to 681136
		5′-GTGCCATTGTTTGGTCAG-3′		
*pheS*	Phenylalanyl-tRNA synthetase subunit alpha	5′-TTTCTTAGGTTTAGGCTTTG-3′	665	406737 to 407813
		5′-CCTTTCGGTTAAATTGTGA-3′		

*Positions correspond to the complete genome sequence of *Leu. mesenteroides* subsp. *mesenteroides* ATCC 8293.

### Recombination in *L. lactis*

The level of linkage disequilibrium between all alleles of the isolates evaluated was high as the calculated *I*_*A*_^S^ was 0.4264 (*p* = 0.000) and significantly different from the *I*_*A*_^S^ value of 0 expected for a population with linkage equilibrium, indicating the genes investigated in this study were close to linkage disequilibrium.

**Figure 1:**
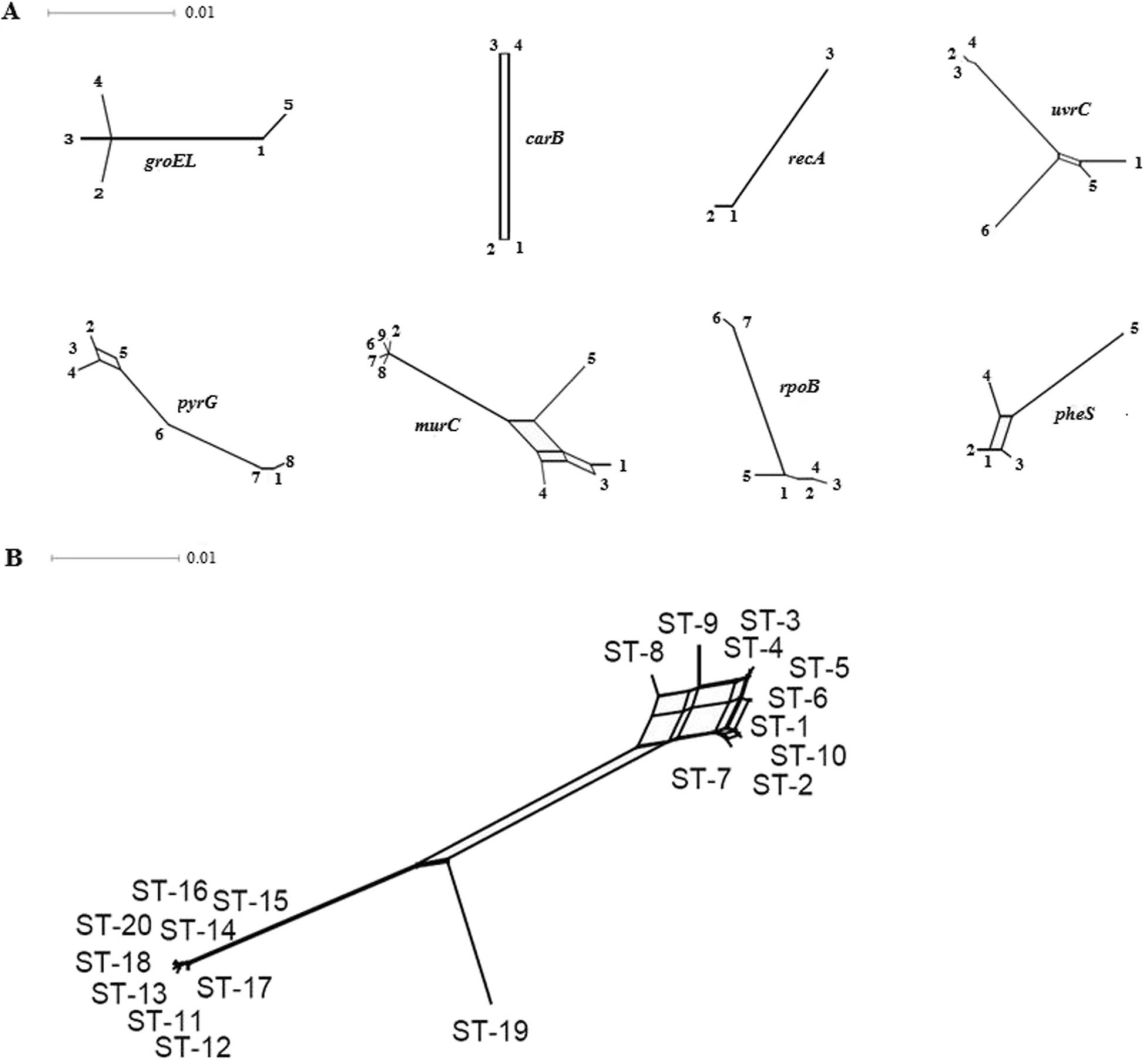
**Split-decomposition analysis based on concatenated sequences of eight housekeeping genes from 50** ***L. lactis *isolates.** Multi-parallelogram formations indicate recombination events. **(A)** Split-decomposition analysis of individual MLST loci. **(B)** Combined split-decomposition analysis of all eight MLST loci.

Split decomposition analysis to examine evolutionary relationships amongst the isolates revealed different structures in the split graphs for all eight loci (Figure 1A). In the split graphs for *murC*, *pheS*, *pyrG* and *uvrC*, the parallelogram-shaped structures detected indicated that intergenic recombination had occurred during the evolution of these four genes. The split graphs obtained for *carB*, *groEL*, *recA* and *rpoB* loci revealed tree-like structures, suggesting that the descent of these genes was clonal and not significantly affected by intergenic recombination. The split graphs of the *recA* and *carB* genes were a polygonal line and columnar respectively because only three (*recA*) or four (*carB*) alleles were analysed.The combined split graph of alleles for all eight MLST loci displayed a network-like structure (Figure 1B). The 20 STs representing all isolates were divided into two main subpopulations and each subpopulation was completely disconnected. ST1 to ST10 were clustered together as one subpopulation, where some parallelogram-shaped groupings were detected. The split graphs for the remaining STs, clustered into a second subpopulation. This suggests that recombination had not occurred between isolates from the two subpopulations, but that intergenic recombination may occur between isolates from the same subpopulation during their evolution. ST19, which contained only isolate MAU80137 from non-traditional dairy production, was clearly disconnected from the others isolates, indicating no recombination had occurred between this isolate and other isolates from either of the two subpopulations.

### Cluster analysis of the MLST data

**Figure 2:**
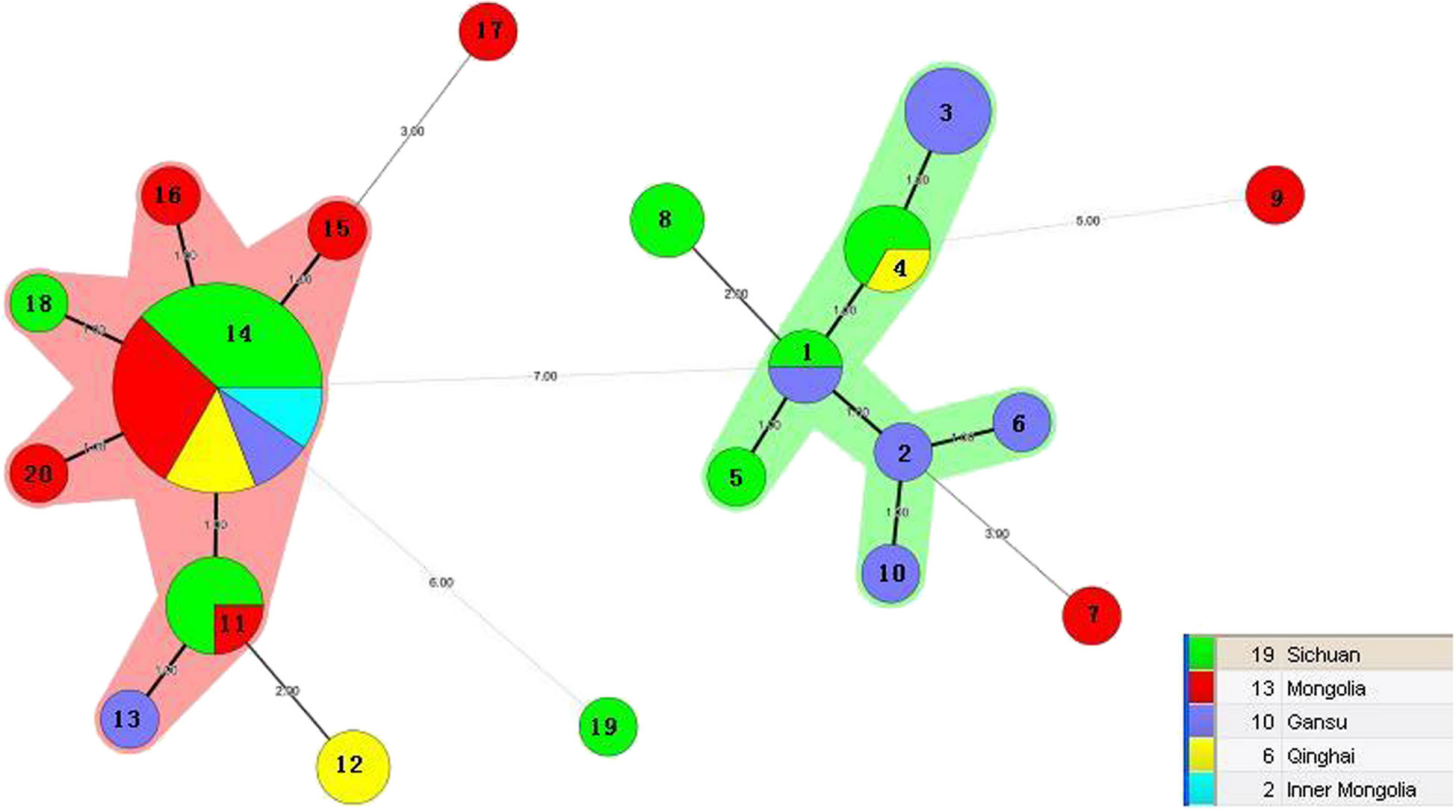
**Minimum-spanning tree analysis of 50** ***L. lactis *isolates based on MLST date according to region.** Each circle indicates a sequence type, the size of the circle is proportional to the number of isolates and the type of line between isolates indicates the strength of the genetic relationship between these isolates (black line = strong relationship, grey line = intermediate relationship and dotted line = weak relationship).

Clustering by region amongst the isolates was evident in the minimum-spanning tree (Figure 2). The 50 *L. lactis* isolates evaluated were assigned to 20 STs that resolved into eight clonal complexes (CCs). Among these CCs, 14 STs were clustered together to form two CCs and there were six singleton STs that could not be assigned to any group.

The largest CC was comprised of ST11, ST13, ST14, ST15, ST16, ST18 and ST20, which included 30 isolates, mainly from Sichuan province and Mongolia. Within this CC (colour-coded pink) ST14 was the predicted primary founder surrounded by single-locus (ST11, ST15, ST16, ST18, and ST20), or two-locus variants (ST13). These STs have been connected by solid black lines indicating they are closely related. The second CC included ST1 to ST6 and ST10, which included 16 isolates mainly from Sichuan and Gansu provinces. ST1 from Sichuan and Gansu province located in the centre of the second clonal complex. Single-locus variants were ST2, ST4 and ST5, which contained isolates from Gansu, Qinghai and Sichuan provinces. Two-locus variants were ST3, ST6 and ST10 and included isolates from Gansu province. ST7, ST8, ST9, ST12, ST17 and ST19 were singletons unlinked to the other CCs. However, they are connected to two primary founders, either ST1 or ST14, by grey or dotted lines, indicating they had a distant relationship with the two predicted ancestors. ST7 and ST8 were two and four-locus variants of ST1 and connected with grey lines. ST12 and ST17 were three- and four-locus variants of ST14 and also connected by grey lines. Isolate IMAU20185 belonging to ST9 was a six-locus variant of ST1 to which it was connected by a dotted line. Isolate IMAU80137 belonging to ST19 was a six-locus variant of ST14 to which it was also connected by a dotted line.

### UPGMA tree based on MLST data

**Figure 3:**
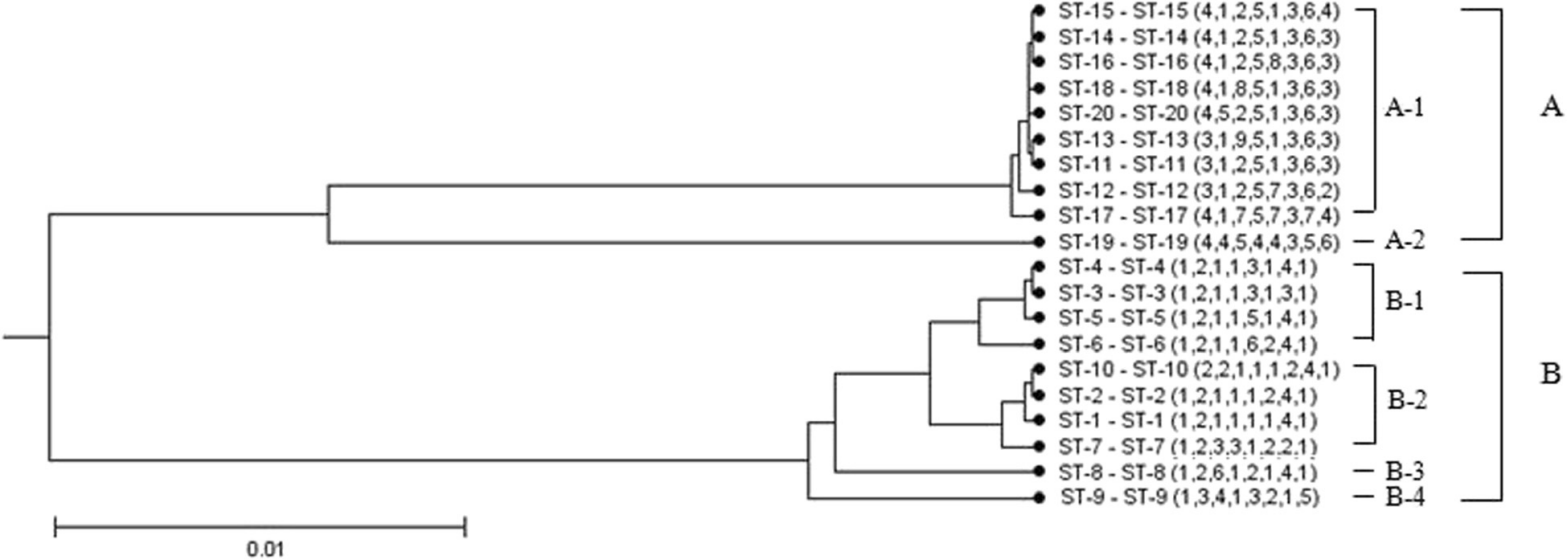
**UPGMA dendrogram showing the genetic relationships between the 20 STs that belong to *L*. *lactis *through MLST typing in this study.** The Phylogenetic tree was produced using START 2.0 software and the UPGMA method. The numbering in the figure refers to the ST. Two major phylogroups were designated as **A** and **B**.

Genetic relatedness amongst the *L. lactis* isolates investigated in this study showed they were well clustered within two major groups, A and B. Group A was comprised of 34 isolates and group B of only 16 isolates. Group A was the better supported group and included two subgroups. Group B was a weakly supported group that included four subgroups (Figure 3). With the exception of ST19, isolates in group A were closely related only differing in two out of the eight loci from the primary founder, ST14. The isolate that belonged to ST19 was a six-locus variant of the primary founder. Isolates in Group B were distantly related and differed in between two and six of the eight loci from the primary founder ST1.

## Discussion

MLST is considered to be the best method for studying molecular epidemiology and population structure of bacteria [29-31]. Although this approach has been developed for several LAB, such as *Lb. plantarum*, *Lb. delbrueckii*, *Lb. casei*, and *O. oeni*[25,26,32], until this study there had been no MLST protocol used for *L. lactis.* In this study, we used MLST with eight housekeeping genes on 50 *L. lactis* isolates from a relatively large geographic area including Mongolia, a number of Chinese Provinces and an Autonomous region. These representative isolates are unique in their diversity of sources and provide the relevant information required for a better understanding of genetic diversity, persistence and movement.

The first step in development of a MLST typing method required analysis of the sequence diversity of eight housekeeping genes from the 50 *L. lactis* isolates under evaluation, to ensure that the MLST protocol had the discriminatory power to type isolates within a single species. The two loci that had low polymorphism, contained three and four polymorphic sites in the *recA* and *carB* loci respectively (Table 1). The low level of biodiversity in *recA* and *carB* suggested they had similar sequences at the species level and would, therefore, have a lower discriminatory ability than the other housekeeping loci used in this study. The remaining six loci, *groEL*, *pheS*, *uvrC*, *rpoB*, *pyrG*, *murC* had more polymorphic sites (between five and nine), suggesting that they would have a good discriminatory ability when used in MLST. A total of 47 polymorphic sites were detected in the eight loci giving a polymorphism rate of 0.88% of the 5,325 nucleotides present. The relatively low number of polymorphic sites suggests that the partial sequences of the eight housekeeping genes were somewhat conserved amongst the 50 *L. lactis* isolates. Although housekeeping genes evolve slowly, however, we still consider that the eight housekeeping genes selected provide sufficient discriminatory power for typing. In our genetic analysis, the eight housekeeping loci had a very low *d*_N_/*d*_S_ ratio (<1), implying strong purifying selection. This was particularly the case in *groEL* where five substitutions were synonymous and the amino acid composition did not change. The *d*_*N*_*/d*_*S*_ ratio was close to zero and similar to that seen by Madslien *et al.*[33], which supports our estimation that the housekeeping loci are all under stabilizing selection [33].

*Leuconostoc lactis* isolates are important industrially in the production of fermented foods. However, their population structure has not been investigated fully before. We used linkage disequilibrium expressed as the index of association (*I*_A_) by the equation, *I*_A_ = V_O_/V_E_ - 1 (V_O_, observed variance; V_E_, expected variance) to investigate population structure. This index of association is a generalised measure of linkage disequilibrium; does not rely on the number of loci analysed; has an expected value of zero if there is no association between loci, i.e. alleles are in linkage equilibrium (free recombination) [34,35]; and was pioneered to describe population structure in *Hordeum spontaneum*[36]. In our study, the value of *I*_A_ and *I*_*A*_^S^ for eight loci were calculated as 1.8 and 0.4264 (*p* = 0.000), respectively. These high values are indicative of a strong clonal population and similar to reports for other bacteria. For example, in *Lb. plantarum*, where seven selected housekeeping genes were analysed and assigned to 17 different STs, the *I*_*A*_^S^ value was 0.444 [37]. In *Bacillus licheniformis*, where six housekeeping loci were analysed from 53 diverse isolates, the value of *I*_*A*_^S^ was 0.4365 [33]. These results are similar to our study on *L. lactis* and, therefore, support our hypothesis that these are clonal populations and that allelic selection is close to linkage disequilibrium.

In general, *Leuconostoc* species are used as starter cultures for dairy fermentations. All isolates initiate lactose fermentation and lactic acid production and here we have shown that some essential housekeeping genes are highly conserved. However, the value of *I*_*A*_^S^ and the number of unique STs reflect the genetic diversity amongst isolates that have each adapted to specialised environments during their evolution. Similar results have been reported for other LAB isolated from dairy products; for example 197 isolates of *Lactococcus lactis* isolated from homemade yogurt were assigned to 72 different STs and their *I*_*A*_^S^ value was 0.3038 [38]. Uniformly, a clonal structure was also found in *Streptococcus thermophilus*, where eight housekeeping loci were analysed from 26 isolates from different dairy products [39]*.*

Split-decomposition analysis based on the allelic profiles of isolates evaluated provided evidence of intraspecies recombination that could play a role in generating genotypic diversity amongst isolates. Parallelogram-shaped structures were commonly found in the split graphs of the partial housekeeping genes (*murC*, *pheS*, *pyrG*, and *uvrC*) and the combined alleles, illustrating recombination had occurred in some MLST loci. Previous studies have provided evidence that recombination could occur frequently in *Leuconostoc* species because mobile elements, such as bacteriophages, genomic islands and transposable elements, were found in the genome sequence [40,41]. In addition, some plasmids from *Leuconostoc* species have been identified [42,43]. In *O. oeni* isolates, a similar recombination phenomenon has been found including the presence of plasmids, bacteriophages and insertion sequences [44-46]*.* Furthermore, the presence of parallelogram-shaped structures were also found in the *ddl*, *pgm* and *recP* split graphs of *O. oeni* isolates [26].

Although this study on the population structure of *L. lactis* has made important steps forward, e.g. the split-decomposition analysis based on concatenated sequences of housekeeping genes (Figure 1), the UPGMA tree based on the MLST data (Figure 3) and the *I*_*A*_^S^ values, we could still not confirm any association between ST and the original source of each isolate. Similar results have been reported in *Lactococcus lactis* and *Lactobacillus sanfranciscensis*, where no significant associations between STs and the various sources of the isolates could be found [47,48]. The absence of such an association in *L. lactis* may be because of the genetic diversity of individual *L. lactis* isolates. At the gene level, MLST analysis indicated two CCs and six singletons. The majority of *L. lactis* isoaltes from dairy products were found in these two CCs; the remaining isolates from various sources including yogurt, kurut, yak’s milk and pickle, were scattered into unique STs. This characterisation was also reflected in the UPGMA dendrogram, with isolates clustering as two groups that could be further divided into several subgroups (Figure 3). These unique STs (ST7, ST8,ST9, ST12, ST17 and ST19) illustrate the genetic diversity within the subspecies.

## Conclusions

A MLST protocol for *L. lactis* isolates, based on eight housekeeping genes and 50 *L. lactis* isolates was developed. In this study, we demonstrated biodiversity, clonal population structure and genetic recombination in the isolates evaluated. All of these isolates could be separated into two distinct groups that had evolved independently from each other, except isolate MAU80137 from ST19. This isolate was the only one from a nontraditional dairy and was only distantly related to all the other isolates analysed. Future work will target other sources of *L. lactis* by examining environmental samples to obtain a better understanding of the evolution and population genetics of *L. lactis*.

## Methods

### *Leuconostoc lactis* isolates

In this study, a total of 50 *L. lactis* isolates, preserved in the Lactic Acid Bacteria Collection Center of the Inner Mongolia Agricultural University (LABCC), were examined and characterised (Additional file 1: Table S1). These isolates originated from various sources including yogurt, kurut, qula and other traditional foods from Mongolia, the P.R. of China Provinces Sichuan, Qinghai, Gansu and the P.R. China Inner Mongolia Autonomous Region. *Leuconostoc lactis* isolate MAU80137 was the only isolate from pickle (Sichuan province). All isolates were identified as *L. lactis* based on standard physiological and biochemical tests, and sequence analysis of the 16S rRNA gene [32,49]. Stock cultures were stored in 10% glycerol at -80°C. Working cultures were retrieved from storage and activated by two subcultures through de Man Rogosa Sharpe (MRS) broth (Becton, Dickinson Co., Sparks, Md., USA). Isolates were incubated at 30°C for 24 h under anaerobic conditions prior to evaluation.

### DNA extraction

Genomic DNA was extracted from all isolates as described previously [50]. Briefly, after overnight incubation in MRS broth at 37°C, the bacterial cells were collected by centrifugation (8,000 × *g*, 3 min, 4°C) and subjected to freeze-thaw cycles for cell lysis. Next, 10% sodium dodecyl sulphate (SDS) and proteinase-K solution (20 mg/ml) were added, mixed well, and incubated in a shaking incubator at 200 rpm and 37°C overnight. This was following by addition of 0.7 M NaCl and 10% cetyltrimethyl ammonium bromide (CTAB) and further incubation at 65°C for 20 minutes. Protein contaminants were removed by the addition of phenol/chloroform/isoamyl alcohol (25/24/1). The DNA was precipitated as a pellet by the addition of an equal volume of ice-cold isopropanol, and then washed in 70% (v/v) ice-cold ethanol and dissolved in sterile ultrapure water. The purity of the extracted DNA was quantified by recording its optical density at 260 and 280 nm, respectively, using a NanoDrop ND-1000 spectrophotometer (NanoDrop Technologies, Wilmington, DE, USA).

### Selection of housekeeping genes for the MLST protocol

Eight loci representing housekeeping genes were selected for MLST on *L. lactis* isolates from those already described from the variable regions of the *L. mesenteroides* subsp. *mesenteroides* ATCC 8293 genome sequence [28]: *pyrG* encoding CTP synthetase (accession no. YP_818007), *rpoB*, encoding DNA-directed RNA polymerase subunit beta (YP_819285.1), *groEL* encoding chaperonin GroEL(YP_819222.1), *recA* encoding recombinase A (YP_818071.1), *uvrC* encoding excinuclease ABC subunit C (YP_818008.1), *carB* encoding carbamoyl phosphate synthase large subunit (YP_818678.1), *murC* encoding UDP-N-acetylmuramate-L-alanine ligase (YP_818192.1), *pheS* encoding phenylalanyl-tRNA synthetase subunit alpha (YP_817936.1).

### Amplification and nucleotide sequencing

Primers for the polymerase chain reaction (PCR) were designed based on the internal fragments of the eight loci using Primers 5 software (Table 2). In this study, the MLST protocol was modified in two ways; firstly, the primers targeting internal fragments of each gene were extended from 450–500 to 500–700 bp and secondly, although MLST protocols generally only use five to seven genes, in this study, eight housekeeping genes were used to analyse the population structure of *L. lactis.* The eight housekeeping gene fragments (*carB*, *groEL*, *murC*, *pheS*, *pyrG*, *recA*, *rpoB*, *uvrC*) were amplified from chromosomal DNA from each isolate using amplification and sequencing primers (Table 2). The *PCR* procedure for the *pyrG*, *carB*, *murC* and *pheS* genes was done under the following conditions: 94°C for 5 sec, 30 cycles of amplification which included 95°C for 60 sec, 50°C for 45 sec, 72°C for 60 sec and then annealing at 72°C for 10 min. PCR for the remaining genes followed the same experimental conditions except for the annealing temperature which was 54°C. PCR reactions were made in a 10 μl reaction mixture containing 0.08 μl Taq polymerase (5 U/μl, Takara, Tokyo), 1 μl 10 × PCR Buffer (Mg^2+^ free), 0.8 μl dNTPs (2.5 mM each), 0.8 μl MgCl_2_ (25 mM), 0.4 μl forward primer (10 μM), 0.4 μl reverse primer (10 μM), 1 μl genomic DNA (10–50 ng/μL), and 5.52 μl dH_2_O. The PCR products were separated by electrophoresis on a 1.2% agarose gel and then visualised using ethidium bromide staining. Sequencing of the PCR products was done by the Shanghai Sangni Biosciences Corporation (Shanghai, China) and the sequences deposited in the GenBank/EMBL databases under accession numbers KJ149820 to KJ150219.

### Data analysis

The sequences obtained for the eight housekeeping genes in the MLST protocol from all isolates were imported into BioNumerics software (version 6.0, Applied-Maths, Sint Maartens-Latem, Belgium) and the *number* of unique alleles per locus obtained. In date analysis, all unique sequences were assigned an allele number and each unique combination of eight allele numbers per isolate was assigned a ST [27]. The guanine-cytosine content, *d*_*N*_*/d*_*S*_ ratio (*d*_*S*_ is the number of synonymous substitutions per synonymous site and *d*_*N*_ is the number of non-synonymous substitutions per non-synonymous site) and the number of polymorphic sites and single nucleotide polymorphisms (SNPs) of the eight housekeeping genes for each isolate were calculated using LIAN-Linkage analysis [51]. The level of linkage disequilibrium between all alleles of the isolates was investigated by determining the standardised index of association (*I*_*A*_^S^) [34]. Phylogenetic trees were constructed by the neighbour-joining (N-J) method in MEGA version 5.0 software (version 5.0, http://www.megasoftware.net). The relationships between MLST STs and analysis of CCs were revealed using eBURST (Based Upon Related Sequence Types) V 3.0 software ( http://eburst.mlst.net). CCs are typically composed of a single predominant genotype with a number of much less common close relatives of that genotype [52]; the isolates of *L. lactis* that shared a minimum of five identical alleles with the central genotype in a given subpopulation were assigned to a different CC. An UPGMA dendrogram was constructed by START 2.0 software using the unweighted pair-group method and the arithmetic average method (UPGMA). The split decomposition was done with SplitsTree and START 2.0 software on the MLST website ( http://eburst.mlst.net/). Minimum-spanning tree analysis of the STs from all isolates was done using Prims’s algorithm in the BioNumerics software according to region and source separation (version 6.0, Applied-Maths, Sint Maartens-Latem, Belgium).

## Competing interests

The authors declare that they have no competing interests.

## Authors’ contributions

Conceived and designed the experiments: TD WJL ZHS HPZ. Performed the experiments: QL HYX YQS. Analyzed the data: ZHS YQS. Contributed reagents/materials/analysis tools: ZHS QL HYX YQS. Wrote the paper: TD HPZ. All authors read and approved the final manuscript.

## Supplementary Material

Additional file 1: Table S1 Allelic profiles of 50 *Leuconostoc lactis* isolates. Click here for file

## References

[B1] De Bruyne K Schillinger U Caroline L Boehringer B Cleenwerck I Vancanneyt M De Vuyst L Franz CM Vandamme P *Leuconostoc holzapfelii* sp. nov., isolated from Ethiopian coffee fermentation and assessment of sequence analysis of housekeeping genes for delineation of *Leuconostoc* species Int J Syst Evol Microbiol 2007 57 Pt 12 2952 2959 18048756 10.1099/ijs.0.65292-0

[B2] Hemme D Foucaud-Scheunemann C *Leuconostoc*, characteristics, use in dairy technology and prospects in functional foods Int Dairy J 2004 14 467 494 10.1016/j.idairyj.2003.10.005

[B3] Ogier JC Casalta E Farrokh C Saïhi A Safety assessment of dairy microorganisms: the *Leuconostoc* genus Int J Food Microbiol 2008 126 3 286 290 10.1016/j.ijfoodmicro.2007.08.012 17897747

[B4] Sharpe ME Garvie EI Tilbury RH Some slime-forming heterofermentative species of the genus *Lactobacillus* Appl Microbiol 1972 23 2 389 397 4259626 10.1128/am.23.2.389-397.1972 PMC380350

[B5] Van Tieghem P Sur la gomme du sucerie (*Leuconostoc mesenteroides*) Ann Sci Nat Bot 1878 7 180 203

[B6] Garvie EI Bergan Separation of species of the genus Leuconostoc and differentiation of the Leuconostocs from other lactic acid bacteria Methods in Microbiology, 16 1984 London: Academic Press 147 178

[B7] Martinez-Murcia AJ Collins MD A phylogenetic analysis of an atypical *Leuconostoc*: description of *Leuconostoc fallax* sp. nov FEMS Microbiol Lett 1991 82 55 60 10.1111/j.1574-6968.1991.tb04839.x 1718816

[B8] Collins MD Samelis J Metaxopoulos J Wallbanks S Taxonomic studies on some *Leuconostoc*-like organisms from fermented sausages: description of a new genus *Weisella* for the *Leuconostoc parame*:renteroides group of species J Appl Bacteriol 1993 75 6 595 603 10.1111/j.1365-2672.1993.tb01600.x 8294308

[B9] Dicks LM Dellaglio F Collins MD Proposal to reclassify *Leuconostoc oenos* as *Oenococcus oeni* [corrig.] gen. nov., comb. nov Int J Syst Bacteriol 1995 45 2 395 397 10.1099/00207713-45-2-395 7537074

[B10] Endo A Okada S Reclassification of the genus *Leuconostoc* and proposals of *Fructobacillus fructosus* gen. nov., comb. nov., *Fructobacillus durionis* comb. nov., *Fructobacillus ficulneus* comb. nov. and *Fructobacillus pseudoficulneus* comb. nov Int J Syst Evol Microbiol 2008 58 Pt 9 2195 2205 18768629 10.1099/ijs.0.65609-0

[B11] Vancanneyt M Zamfir M de Wachter M Cleenwerck I Hoste B Rossi F Dellaglio F de Vuyst L Swings J Reclassification of *Leuconostoc argentinum* as a later synonym of *Leuconostoc lactis* Int J Syst Evol Microbiol 2006 56 213 216 10.1099/ijs.0.63898-0 16403889

[B12] Jeong SH Lee SH Jung JY Choi EJ Jeon CO Microbial succession and metabolite changes during long-term storage of Kimchi J Food Sci 2013 78 5 M763 769 10.1111/1750-3841.12095 23550842

[B13] Ehrmann MA Freiding S Vogel RF *Leuconostoc palmae* sp. nov., a novel lactic acid bacterium isolated from palm wine Int J Syst Evol Microbiol 2009 59 Pt 5 943 947 19406772 10.1099/ijs.0.005983-0

[B14] Lee SH Park MS Jung JY Jeon CO *Leuconostoc miyukkimchii* sp. nov., isolated from brown algae (Undaria pinnatifida) kimchi Int J Syst Evol Microbiol 2012 62 Pt 5 1098 1103 10.1099/ijs.0.032367-0 21705441

[B15] Sabat AJ Budimir A Nashev D Sá-Leão R van Dijl J Laurent F Grundmann H Friedrich AW Overview of molecular typing methods for outbreak detection and epidemiological surveillance Euro Surveill 2013 18 4 20380 23369389 10.2807/ese.18.04.20380-en

[B16] Pérez G Cardell E Zárate V Random amplified polymorphic DNA analysis for differentiation of *Leuconostoc mesenteroides* subspecies isolated from Tenerife cheese Lett Appl Microbiol 2002 34 2 82 85 10.1046/j.1472-765x.2002.01050.x 11849499

[B17] Cibik R Lepage E Talliez P Molecular diversity of *Leuconostoc mesenteroides* and *Leuconostoc citreum* isolated from traditional french cheeses as revealed by RAPD fingerprinting, 16S rDNA sequencing and 16S rDNA fragment amplification Syst Appl Microbiol 2000 23 2 267 278 10.1016/S0723-2020(00)80014-4 10930080

[B18] Villani F Moschetti G Blaiotta G Coppola S Characterization of strains of *Leuconostoc mesenteroides* by analysis of soluble whole-cell protein pattern, DNA fingerprinting and restriction of ribosomal DNA J Appl Microbiol 1997 82 5 578 588 10.1111/j.1365-2672.1997.tb03588.x 9172399

[B19] Alegría Á Delgado S Flórez AB Mayo B Identification, typing, and functional characterization of *Leuconostoc* spp. strains from traditional, starter-free cheeses Dairy Sci Technol 2013 93 657 673 10.1007/s13594-013-0128-3

[B20] Nieto-Arribas P Seseña S Poveda JM Palop L Cabezas L Genotypic and technological characterization of *Leuconostoc* isolates to be used as adjunct starters in Manchego cheese manufacture Food Microbiol 2010 27 85 93 10.1016/j.fm.2009.08.006 19913697

[B21] Sánchez JI Martínez B Rodríguez A Rational selection of *Leuconostoc* strains for mixed starters based on the physiological biodiversity found in raw milk fermentations Int J Food Microbiol 2005 105 377 387 10.1016/j.ijfoodmicro.2005.04.025 16085331

[B22] Vihavainen EJ Björkroth KJ Diversity of *Leuconostoc gasicomitatum* associated with meat spoilage Int J Food Microbiol 2009 136 1 32 36 10.1016/j.ijfoodmicro.2009.09.010 19836091

[B23] Björkroth KJ Geisen R Schillinger U Weiss N De Vos P Holzapfel WH Korkeala HJ Vandamme P Characterization of *Leuconostoc gasicomitatum* sp. nov., associated with spoiled raw tomato-marinated broiler meat strips packaged under modified-atmosphere conditions Appl Environ Microbiol 2000 66 9 3764 3772 10.1128/AEM.66.9.3764-3772.2000 10966388 PMC92218

[B24] Maiden MC Bygraves JA Feil E Morelli G Russell JE Urwin R Zhang Q Zhou J Zurth K Caugant DA Feavers IM Achtman M Spratt BG Multilocus sequence typing: a portable approach to the identification of clones within populations of pathogenic microorganisms Proc Natl Acad Sci U S A 1998 95 6 3140 3145 10.1073/pnas.95.6.3140 9501229 PMC19708

[B25] Tanigawa K Watanabe K Multilocus sequence typing reveals a novel subspeciation of *Lactobacillus delbrueckii* Microbiol 2011 157 727 738 10.1099/mic.0.043240-0 21178164

[B26] De Las RB Marcobal A Muñoz R Allelic diversity and population structure in *Oenococcus oeni* as determined from sequence analysis of housekeeping genes Appl Environ Microbiol 2004 70 12 7210 7219 10.1128/AEM.70.12.7210-7219.2004 15574919 PMC535203

[B27] Bilhère E Lucas PM Claisse O Lonvaud-Funel A Multilocus sequence typing of *Oenococcus oeni*: detection of two subpopulations shaped by intergenic recombination Appl Environ Microbiol 2009 75 5 1291 1300 10.1128/AEM.02563-08 19114515 PMC2648160

[B28] Makarova K Slesarev A Wolf Y Sorokin A Mirkin B Koonin E Pavlov A Pavlova N Karamychev V Polouchine N Shakhova V Grigoriev I Lou Y Rohksar D Lucas S Huang K Goodstein DM Hawkins T Plengvidhya V Welker D Hughes J Goh Y Benson A Baldwin K Lee JH Díaz-Muñiz I Dosti B Smeianov V Wechter W Barabote R Comparative genomics of the lactic acid bacteria Proc Natl Acad Sci U S A 2006 103 42 15611 15616 10.1073/pnas.0607117103 17030793 PMC1622870

[B29] Liang J Ducatelle R Pasmans F Smet A Haesebrouck F Flahou B Multilocus sequence typing of the porcine and human gastric pathogen *Helicobacter suis* J Clin Microbiol 2013 51 3 920 926 10.1128/JCM.02399-12 23303499 PMC3592083

[B30] Baldo L Dunning Hotopp JC Jolley KA Bordenstein SR Biber SA Choudhury RR Hayashi C Maiden MC Tettelin H Werren JH Multilocus sequence typing system for the endosymbiont *Wolbachia pipientis* Appl Environ Microbiol 2006 72 11 7098 7110 10.1128/AEM.00731-06 16936055 PMC1636189

[B31] Bisharat N Cohen DI Harding RM Falush D Crook DW Peto T Maiden MC Hybrid Vibrio vulnificus Emerg Infect Dis 2005 11 1 30 35 10.3201/eid1101.040440 15705319 PMC3294331

[B32] Diancourt L Passet V Chervaux C Garault P Smokvina T Brisse S Multilocus sequence typing of *Lactobacillus casei* reveals a clonal population structure with low levels of homologous recombination Appl Environ Microbiol 2007 73 20 6601 6611 10.1128/AEM.01095-07 17704267 PMC2075077

[B33] Madslien EH Olsen JS Granum PE Blatny JM Genotyping of B. licheniformis based on a novel multi-locus sequence typing (MLST) scheme BMC Microbiol 2012 12 230 10.1186/1471-2180-12-230 23051848 PMC3492095

[B34] Suerbaum S Lohrengel M Sonnevend A Ruberg F Kist M Allelic diversity and recombination in *Campylobacter jejuni* J Bacteriol 2001 183 8 2553 2559 10.1128/JB.183.8.2553-2559.2001 11274115 PMC95172

[B35] Huson DH SplitsTree: analyzing and visualizing evolutionary data Bioinform 1998 14 68 73 10.1093/bioinformatics/14.1.68 9520503

[B36] Brown AH Feldman MW Nevo E Multilocus structure of natural populations of HORDEUM SPONTANEUM Genetics 1980 96 2 523 536 17249067 10.1093/genetics/96.2.523 PMC1214315

[B37] De Las RB Marcobal A Muñoz R Development of a multilocus sequence typing method for analysis of *Lactobacillus plantarum* strains Microbiol 2006 152 Pt 1 85 93 10.1099/mic.0.28482-0 16385118

[B38] Xu H Sun Z Liu W Yu J Song Y Lv Q Zhang J Shao Y Menghe B Zhang H Multilocus sequence typing of Lactococcus lactis from naturally fermented milk foods in ethnic minority areas of China J Dairy Sci 2014 doi:10.3168/jds.2013-7738 10.3168/jds.2013-7738 24612812

[B39] Delorme C Bartholini C Bolotine A Ehrlich SD Renault P Emergence of a cell wall protease in the *Streptococcus thermophilus* population Appl Environ Microbiol 2010 76 2 451 460 10.1128/AEM.01018-09 19915034 PMC2805209

[B40] Meslier V Loux V Renault P Genome sequence of *Leuconostoc pseudomesenteroides* strain 4882, isolated from a dairy starter culture J Bacteriol 2012 194 23 696 712 10.1128/JB.01696-12 PMC3497538 23144391

[B41] Nam SH Choi SH Kang A Kim DW Kim RN Kim A Park HS Genome sequence of *Leuconostoc argentinum* KCTC 3773 J Bacteriol 2010 192 24 6490 6491 10.1128/JB.01148-10 20952569 PMC3008539

[B42] Chang JY Chang HC Identification of a replicon from pCC3, a cryptic plasmid from *Leuconostoc citreum* C4 derived from kimchi, and development of a new host-vector system Biotechnol Lett 2009 31 5 685 696 10.1007/s10529-009-9912-9 19142587

[B43] Jeong SJ Park JY Lee HJ Kim JH Characterization of pFMBL1, a small cryptic plasmid isolated from *Leuconostoc mesenteroides* SY2 Plasmid 2007 57 3 314 323 10.1016/j.plasmid.2006.09.003 17084452

[B44] Brito L Pavela H Presence and analysis of large plasmids in *Oenococcus oeni* Plasmid 1999 41 260 267 10.1006/plas.1999.1397 10366531

[B45] Ze’-Ze’ L Tenreiro R Pavela H The *Oenococcus oeni* genome: physical and genetic mapping of a strain GM and comparison with the genome of a “divergent” strain, PSU-1 Microbiol 2000 146 3195 3204 10.1099/00221287-146-12-3195 11101677

[B46] Zavaleta AI Martı’nez-Murcia AJ Rodrı’guez-Varela F 16S-23S rDNA intergenic sequences indicate that *Leuconostoc oenos* is phylogenetically homogeneous Microbiol 1996 142 2105 2114 10.1099/13500872-142-8-2105 8760923

[B47] Picozzi C Bonacina G Vigentini I Foschino R Genetic diversity in Italian *Lactobacillus sanfranciscensis* strains assessed by multilocus sequence typing and pulsed-field gel electrophoresis analyses Microbiol 2010 156 2035 2045 10.1099/mic.0.037341-0 20360177

[B48] Passerini D Beltramo C Coddeville M Quentin Y Ritzenthaler P Daveran-Mingot M-L Le Bourgeois P Genes but not genomes reveal bacterial domestication of *Lactococcus lactis* PLoS ONE 2010 5 12 e15306 10.1371/journal.pone.0015306 21179431 PMC3003715

[B49] Bao Q Liu W Yu J Wang W Qing M Chen X Wang F Zhang J Zhang W Qiao J Sun T Zhang H Isolation and identification of cultivable lactic acid bacteria in traditional yak milk products of Gansu Province in China J Gen Appl Microbiol 2012 58 2 95 105 10.2323/jgam.58.95 22688240

[B50] Dan T Cheng X Bao QH Liu WJ Zhang HP Effect of L-Threonine concentrations on acetaldehyde production and *glyA* gene expression in fermented milk by *Streptococcus thermophilus* Food Biotechnol 2012 26 3 280 292 10.1080/08905436.2012.699204

[B51] Smith JM Smith NH O’Rourke M Spratt BG How clonal are bacteria? Proc Natl Acad Sci U S A 1993 90 10 4384 4388 10.1073/pnas.90.10.4384 8506277 PMC46515

[B52] Feil EJ Cooper JE Grundmann H Robinson DA Enright MC Berendt T Peacock SJ Smith JM Murphy M Spratt BG Moore CE Day NP How clonal is *Staphylococcus aureus*? J Bacteriol 2003 185 3307 3316 10.1128/JB.185.11.3307-3316.2003 12754228 PMC155367

