# Supplementary material for: A novel multi-locus sequence typing (MLST) protocol for Leuconostoc lactis isolates from traditional dairy products in China and Mongolia
Source: BMC Microbiol. 2014 Jun 9;14:150. doi: 10.1186/1471-2180-14-150 (PMC4063691; doi:10.1186/1471-2180-14-150)
Supplement: Additional file 1: Table S1 — Allelic profiles of 50 Leuconostoc lactis isolates. [file 1471-2180-14-150-S1.doc]

**Table S1 - Allelic profiles of 50 *Leuconostoc lactis* isolates**

| **Strain no.** | **Type of product and collection place** | **Year of isolation** | **ST** | **Allele** | | | | | | | | **UPGMAssubgroup** |
| --- | --- | --- | --- | --- | --- | --- | --- | --- | --- | --- | --- | --- |
|  |  |  |  | ***carB*** | ***groEL*** | ***murC*** | ***pheS*** | ***pyrG*** | ***recA*** | ***rpoB*** | ***uvrC*** |  |
| IMAU10446 | Yoghourt,Inner Mongolia | 2009 | 14 | 4 | 1 | 2 | 5 | 1 | 3 | 6 | 3 | A |
| IMAU11004 | Yoghourt, Inner Mongolia | 2009 | 14 | 4 | 1 | 2 | 5 | 1 | 3 | 6 | 3 |  |
| IMAU20181 | Yoghourt, Mongolia | 2009 | 14 | 4 | 1 | 2 | 5 | 1 | 3 | 6 | 3 |  |
| IMAU20182 | Yoghourt, Mongolia | 2009 | 14 | 4 | 1 | 2 | 5 | 1 | 3 | 6 | 3 |  |
| IMAU20184 | Yoghourt, Mongolia | 2009 | 14 | 4 | 1 | 2 | 5 | 1 | 3 | 6 | 3 |  |
| IMAU20186 | Yoghourt, Mongolia | 2009 | 14 | 4 | 1 | 2 | 5 | 1 | 3 | 6 | 3 |  |
| IMAU20187 | Yoghourt, Mongolia | 2009 | 14 | 4 | 1 | 2 | 5 | 1 | 3 | 6 | 3 |  |
| IMAU20199 | Yoghourt, Mongolia | 2009 | 14 | 4 | 1 | 2 | 5 | 1 | 3 | 6 | 3 |  |
| IMAU40032 | Kurut, Qinghai | 2005 | 14 | 4 | 1 | 2 | 5 | 1 | 3 | 6 | 3 |  |
| IMAU40043 | Kurut, Qinghai | 2005 | 14 | 4 | 1 | 2 | 5 | 1 | 3 | 6 | 3 |  |
| IMAU40120 | Kurut, Qinghai | 2005 | 14 | 4 | 1 | 2 | 5 | 1 | 3 | 6 | 3 |  |
| IMAU80256 | Yoghourt, Sichuan | 2009 | 14 | 4 | 1 | 2 | 5 | 1 | 3 | 6 | 3 |  |
| IMAU80258 | Yak milk, Sichuan | 2009 | 14 | 4 | 1 | 2 | 5 | 1 | 3 | 6 | 3 |  |
| IMAU80360 | Yak milk, Sichuan | 2009 | 14 | 4 | 1 | 2 | 5 | 1 | 3 | 6 | 3 |  |
| IMAU80376 | Milk, Sichuan | 2009 | 14 | 4 | 1 | 2 | 5 | 1 | 3 | 6 | 3 |  |
| IMAU80390 | Kurut, Sichuan | 2009 | 14 | 4 | 1 | 2 | 5 | 1 | 3 | 6 | 3 |  |
| IMAU80431 | Yak milk, Sichuan | 2009 | 14 | 4 | 1 | 2 | 5 | 1 | 3 | 6 | 3 |  |
| IMAU80449 | Yogurt whey, Sichuan | 2009 | 14 | 4 | 1 | 2 | 5 | 1 | 3 | 6 | 3 |  |
| IMAU80456 | Yak milk, Sichuan | 2009 | 14 | 4 | 1 | 2 | 5 | 1 | 3 | 6 | 3 |  |
| IMAU80711 | Kurut, Gansu | 2009 | 14 | 4 | 1 | 2 | 5 | 1 | 3 | 6 | 3 |  |
| IMAU80805 | Yak milk, Gansu | 2009 | 14 | 4 | 1 | 2 | 5 | 1 | 3 | 6 | 3 |  |
| IMAU20272 | Yoghourt, Mongolia | 2009 | 11 | 3 | 1 | 2 | 5 | 1 | 3 | 6 | 3 |  |
| IMAU80375 | Yak milk, Sichuan | 2009 | 11 | 3 | 1 | 2 | 5 | 1 | 3 | 6 | 3 |  |
| IMAU80378 | Yak milk, Sichuan | 2009 | 11 | 3 | 1 | 2 | 5 | 1 | 3 | 6 | 3 |  |
| IMAU80380 | Yak milk, Sichuan | 2009 | 11 | 3 | 1 | 2 | 5 | 1 | 3 | 6 | 3 |  |
| IMAU80253 | Qula, Sichuan | 2009 | 18 | 4 | 1 | 8 | 5 | 1 | 3 | 6 | 3 |  |
| IMAU20191 | Yoghourt, Mongolia | 2009 | 16 | 4 | 1 | 2 | 5 | 8 | 3 | 6 | 3 |  |
| IMAU20192 | Yoghourt, Mongolia | 2009 | 15 | 4 | 1 | 2 | 5 | 1 | 3 | 6 | 4 |  |
| IMAU20188 | Yoghourt, Mongolia | 2009 | 20 | 4 | 5 | 2 | 5 | 1 | 3 | 6 | 3 |  |
| IMAU80821 | Yak milk, Gansu | 2009 | 13 | 3 | 1 | 9 | 5 | 1 | 3 | 6 | 3 |  |
| IMAU80324 | Yak milk, Sichuan | 2009 | 1 | 1 | 2 | 1 | 1 | 1 | 1 | 4 | 1 | B |
| IMAU80818 | Qula, Gansu | 2009 | 1 | 1 | 2 | 1 | 1 | 1 | 1 | 4 | 1 |  |
| IMAU80716 | Yogurt whey, Gansu | 2009 | 2 | 1 | 2 | 1 | 1 | 1 | 2 | 4 | 1 |  |
| IMAU80654 | Qula, Gansu | 2009 | 3 | 1 | 2 | 1 | 1 | 3 | 1 | 3 | 1 |  |
| IMAU80656 | Qula, Gansu | 2009 | 3 | 1 | 2 | 1 | 1 | 3 | 1 | 3 | 1 |  |
| IMAU80657 | Qula, Gansu | 2009 | 3 | 1 | 2 | 1 | 1 | 3 | 1 | 3 | 1 |  |
| IMAU50090 | Kurut, Qinghai | 2005 | 4 | 1 | 2 | 1 | 1 | 3 | 1 | 4 | 1 |  |
| IMAU80326 | Yak milk, Sichuan | 2009 | 4 | 1 | 2 | 1 | 1 | 3 | 1 | 4 | 1 |  |
| IMAU80377 | Yak milk, Sichuan | 2009 | 4 | 1 | 2 | 1 | 1 | 3 | 1 | 4 | 1 |  |
| IMAU80408 | Qula, Sichuan | 2009 | 5 | 1 | 2 | 1 | 1 | 5 | 1 | 4 | 1 |  |
| IMAU80516 | Cream, Gansu | 2009 | 6 | 1 | 2 | 1 | 1 | 6 | 2 | 4 | 1 |  |
| IMAU80589 | Milk, Gansu | 2009 | 10 | 2 | 2 | 1 | 1 | 1 | 2 | 4 | 1 |  |
| IMAU20190 | Yoghourt, Mongolia | 2009 | 7 | 1 | 2 | 3 | 3 | 1 | 2 | 2 | 1 | B |
| IMAU80288 | Yak milk, Sichuan | 2009 | 8 | 1 | 2 | 6 | 1 | 2 | 1 | 4 | 1 | B |
| IMAU80289 | Yak milk, Sichuan | 2009 | 8 | 1 | 2 | 6 | 1 | 2 | 1 | 4 | 1 |  |
| IMAU20185 | Yoghourt, Mongolia | 2009 | 9 | 1 | 3 | 4 | 1 | 3 | 2 | 1 | 5 | B |
| IMAU40034 | Kurut, Qinghai | 2005 | 12 | 3 | 1 | 2 | 5 | 7 | 3 | 6 | 2 | A |
| IMAU40035 | Kurut, Qinghai | 2005 | 12 | 3 | 1 | 2 | 5 | 7 | 3 | 6 | 2 |  |
| IMAU20662 | Yoghourt, Mongolia | 2009 | 17 | 4 | 1 | 7 | 5 | 7 | 3 | 7 | 4 | A |
| IMAU80137 | Kraut, Sichuan | 2008 | 19 | 4 | 4 | 5 | 4 | 4 | 3 | 5 | 6 | A |

*All the strains were isolated in our laboratory.
